# A Disturbance Rejection Framework for the Study of Traditional Chinese Medicine

**DOI:** 10.1155/2014/787529

**Published:** 2014-06-04

**Authors:** Han Zhang, Yan Sun, Zhiqiang Gao, Yueqi Wang

**Affiliations:** ^1^Department of Electrical and Computer Engineering, Cleveland State University, Cleveland, OH 44115, USA; ^2^School of Pre-Clinical Medicine, Beijing University of Chinese Medicine, Beijing 100029, China

## Abstract

The traditional Chinese medicine (TCM) is explained in the language of engineering cybernetics (EC), an engineering science with the tradition of rigor and long history of practice. The inherent connection is articulated between EC, as a science of interrelations, and the Chinese conception of Wuxing. The combined cybernetic model of Wuxing seems to have significant explaining power for the TCM and could potentially facilitate better communications of the insights of the TCM to the West. In disturbance rejection, an engineering concept, a great metaphor, is found to show how the TCM is practiced, using the liver cancer pathogenesis and treatment as a case study. The results from a series of experimental studies seem to lend support to the cybernetic model of Wuxing and the principles of disturbance rejection.

## 1. Introduction

Holistic, self-contained, and independent developed over millenniums, traditional Chinese medicine (TCM) is the embodiment of a cosmic view, rooted in Yin-Yang and Wuxing, in the inner working of human bodies. The TCM traces its origin to the Yellow Emperor's Canon of Medicine, which systematically builds the basic tenants of Chinese medicine in the language of Yin-Yang and Wuxing. Westerners may be familiar with the term Yin-Yang but not with the term Wuxing, whose standard translation is “five elements,” which is, unfortunately, a bit misleading. In Wuxing the Chinese try to describe the characteristics of changes in matters, rather than the matters themselves, using the oldest tool of the Chinese thinkers: analogy. In philosophy and in medicine, the Chinese are more interested in how things change, rather than the things in themselves. The Yin and Yang are two opposing forces behind all changes in nature and in human body. Likewise, in Wuxing, water, fire, wood, metal, and earth are used as analogies to symbolize the mutual relationships of promotion and restriction. Take, for example, water, in nature it nourishes the wood and restricts fire; in human body it mirrors kidney, promotes liver (wood), and restricts heart (fire). It is through such analogies Wuxing is systematically used to explain human physiology and pathology [1, 2]. Starting with the Yellow Emperor's Canon of Medicine, Wuxing has become the cornerstone of the TCM that has evolved over the millenniums and is still the basis of the TCM, that is, widely practiced today in China and beyond.

There is little doubt in the effectiveness of some of the TCM practices even in the West, as the wide acceptance of acupuncture, for example, attests. It is also widely recognized, however, that due to the historical and cultural differences, the tenants of TCM are often considered rather abstract and vague and are prone to misinterpretations, in the context of Western science. The values of TCM and the desire to understand it in the language of Western science have occupied the minds of scholars of many generations, East and West.

Much work has been done to quantify the TCM using the methodologies of modern science, including those from anatomy, physiology, pathology, pharmacology, biochemistry, cell biology, and molecular biology. Much progress has been made that gives us new insight into the workings of human body, disease, and treatments, all based on the TCM. The limitations of such line of investigation, however, are also evident in dissecting the holistic TCM using the reductive approach of the modern science. Physiologically and pathologically, a living body is not just a collection of molecules, cells, tissues, and organs, governed, but it is a complex whole governed by the laws of interrelationships among them and between them and their environments Precisely such interrelationships are front and central in the conceptions of the TCM and have been largely beyond the grasp of quantitative studies of the Western science. The missing link, it seems, is the science of interrelationships, to which we now turn.

It is well known in cell biology that a cell, the basic building block of life, constantly communicates with its environment and other cells in what is called cell signaling, which describes the generation, transmission, and reception of biological signals as well as the sequence of actions they trigger. Such “signaling” is commonly seen in engineering systems and is the subject of study in separate engineering disciplines such as signal processing, control, and communication. Such commonalities behind the biological and engineering mechanisms did not escape the mind of the American mathematician Wiener, who in 1948 wrote the landmark book titled “Cybernetics: or Control and Communication in the Animal and the Machine” [3], followed by Tsien's book of “Engineering Cybernetics” in 1954 based on which a whole new engineering science of mutual relationships was born [4].

Unlike other natural sciences of matter, energy, heat, and so forth, cybernetics, according to Tsien, is a general science of “the qualitative aspects of the interrelations among various components of a system and the synthetic behavior of the complete mechanism.” Sounds like Wuxing? For example, in Wuxing one is concerned with the balance of the whole system, natural or human; likewise, in cybernetics one is mesmerized by the quality called stability, which is found not in individual components of a system but in how they are connected, or related, to each other. This shared concern by Wuxing and cybernetics on the holistic behavior rather than the material parts of system gives rise to the hope that, perhaps, the ideas of the TCM can be exposited through the language of cybernetics, a basic tenant of this paper. Instead of the five elements, we use the phase the cybernetic model of Wuxing in this paper to emphasize the connection and commonality in Wuxing and cybernetics.

Engineering cybernetics (EC), like the TCM, is guided by the general principles but is also very pragmatic. It aims at, according to Tsien, “those parts of the broad science of cybernetics which have direct engineering applications in designing controlled or guided systems” [4]. After sixty years of furious developments, engineering cybernetics has become a well-established field of scientific study with rigorous mathematical foundation and a set of extremely effective tools to guide the engineering practice.

Similar to the concept of Wuxing and its embodiments in the practice of TCM, EC has as primary goal the establishment and retention of balance in a dynamic system in the presence of internal and external disturbances. A particular example is the principle of active disturbance rejection control (ADRC) and its various embodiments in different domains of engineering [5–7]. The concepts and tools like ADRC give researchers a much needed vocabulary and methodology to study the TCM, where the diseases are caused by internal and external disturbances and the treatments can be generally seen as various means of disturbance rejection.

The human body is both a wonder and a mystery. This paper takes a small step to clarify the complexity by explaining the principle of Wuxing, which is central to the TCM, in the language of EC and ADRC. Interestingly perhaps to the Western scholars, Wuxing gives a holistic view of the complex system of human body, particularly in how the vital systems interact with each other. By understanding such interrelationship, described in the language of EC, the ADRC framework is then borrowed to explain how the disturbance rejection is accomplished in the TCM. The congenial connection between the system concepts and tools of cybernetics and the Ying-Yang and Wuxing in the TCM gives us hope to quantify and standardize the teachings of the TCM on a rigorous scientific foundation. To this end, the paper is organized as follows: the disturbance rejection paradigm in engineering and its connection to the TCM are explored in Section 2; the cybernetic model of Wuxing and disturbance rejection in the context of TCM are discussed in Section 3, followed by a case study in Section 4, using the liver cancer treatment as an example. Finally, some concluding remarks are given in Section 5.

## 2. Disturbance Rejection in Engineering Cybernetics

Biological organisms are large and complex systems that interact with each other and with the environment. In the context of the TCM, it is the same dynamic balance that one seeks among the organs in the body and between the body and nature in which it resides. Such balance is the foundation of any bio-organisms and it is explained in the vocabulary of the Yin-Yang and Wuxing. It was recognized universally that an organism always possesses internally a self-regulation mechanism, whether it is called the Yin-Yang balance in the East or homeostasis in the West. It is also evident to all that the destruction of such balance, regardless of the causes, invariably leads to the destruction of bodily functions and therefore health. The restoration of such balance is the aim of both Western medicine and the TCM. What makes the communication between the two difficult is the incompatibility in the languages that reflects the incompatibility in the way of thinking. The science of cybernetics offers a new path by which a quantitative study of the TCM can be explored.

### 2.1. Cybernetics

The engineering practice of control and communication long proceeded Wiener's book of 1948. The name cybernetics was chosen because of Wiener's wish “to recognize that the first significant paper on feedback mechanism is an article on governors, which was published by Clerk Maxwell in 1868 and that governor is derived from a Latin corruption of *κυβερνήτης*” [3]. In cybernetics, Wiener initiated a new science that was previously in “a no-man's land between the established fields;” it overlaps with “pure mathematics, statistics, electrical engineering, and neurophysiology.” By the time the second edition was published in 1961, the field has become “an existing science” and “a whole discipline for the engineer, for the physiologist, for the psychologist, and for the sociologist” [8]. This is because all these various kinds of specialties are concerned with interrelations among components within a system. These interrelations are important in studying and modifying system behaviors, particularly the balanced behaviors that are the most desirable.

The study of interrelations took a drastic turn, with increasingly more significant practicality, after the invention of feedback control in servomechanisms and communication engineering, where interrelations among process variables are modified intentionally to achieve desired system characteristics. Causality is seen as linear in Western science for centuries. But now, with cybernetics, scholars began to contemplate a new kind of causality, that is, “circular,” much like Wuxing in the TCM where the key entities in a system, whether in nature or human body, are mutually dependent. But unlike Wuxing and Yin-Yang, the study of cybernetics led to quantitative sciences such as information theory and control theory, which are known for their mathematical rigor and clarity in thinking, the qualities solely lacked in the texts of the TCM.

### 2.2. Engineering Cybernetics

Our research is therefore largely motivated by combining the methodology of cybernetics and the TCM in order to make the transition in the study of the TCM from qualitative to quantitative, along the line of EC suggested by Tsien six decades ago.

Engineering cybernetics is a branch of engineering science founded by H.S. Tsien in his ground breaking book “Engineering Cybernetics” of 1954. Its aim is “to study those parts of the broad science of cybernetics which have direct engineering applications in designing controlled or guided systems.” In EC the study of cybernetics takes a turn from qualitative to quantitative and the book of Tsien “anticipated much of the development after 1954” [9]. After furious developments in the cold war era, fueled by the space and arms race, a great deal of resources have been put into use to further develop the science of cybernetics, giving it a well-established mathematical foundation and the tradition of rigor. Today, feedback control theory, in particular, is highly developed in separate areas of linear and nonlinear system theory, multivariable control, adaptive control, estimating, system identification, and so forth. The paradigm of modeling and model-based control design has firmly established itself as the dominant paradigm in the modern era.

Another line of investigation, completely different from the model-based paradigm, in EC is perhaps a better fit for the study of the TCM. With foresight, Tsien pointed out in 1954 that control design cannot be solely dependent on the accurate mathematical model of the physical systems, because of the complexity and uncertainties involved in trying to capture the system dynamics, which constantly changes during operation. Such challenge is especially daunting in human physiology where accurate information of system dynamics is difficult to obtain and, even when it is obtained, it is unreliable. This leaves with us but one alternative, to which we now turn.

### 2.3. The Disturbance Rejection Paradigm

The disturbance rejection paradigm in EC originated in the landmark paper of Professor Han of 1989 where he carefully distinguishes the theory of engineering cybernetics from that of modern control theory [10]. He argues that a good control design is possible in the absence of global mathematical model because the information needed can be obtained locally in time and space. Han subsequently spend the next twenty years devising such a new mechanism of control where a simplistic dynamic model is assumed and the rest of dynamics, internal and external, is treated as disturbance to be estimated and cancelled in real time [5, 10]. The new paradigm successfully combined the ancient idea of the South-Pointing Chariot with the modern concepts of state and state observers, leading to the ground breaking principles and technologies of ADRC [6, 7, 11]. ADRC as a disruptive technology has been successfully validated in many industrial applications such as motion control, process control, robotics, automobile, and high energy physics [5, 12–16]. It has also been adopted by the industry giant, Texas Instruments, in a new line of DSP chips.

In ADRC, engineering scientists finally find a powerful weapon to systematically deal with vast amount of uncertainties in the physical world. This is of crucial importance in engineering cybernetics and, by extension, to the study of the TCM, because, as argued in [6], most problems of feedback can be seen as the problem of disturbance rejection. By exploring this common ground behind all dynamic systems, engineering or otherwise, we seek to connect the grand cosmic view and the heuristic practice in the TCM with the rigor and clarity of engineering cybernetics.

In the following section, the problem of the TCM will be explored in the framework of disturbance rejection where the engineering cybernetics approach is applicable.

## 3. Disturbance Rejection and the TCM

The life science of the East, to which the TCM belongs, exhibits its unique perspective on many fronts: the ecological view of medicine where the inner workings of nature and man mirror each other; the view of life as self-adjusting and self-stabilizing; the view of medical treatment, that is, seasonal, local and individual; and, finally, the goal of maintaining the Yin-Yang balance and the means for its restoration in the treatments of ailments. It is a great challenge to see such a distinct cosmic view through the lenses of modern science where rigorous analysis described in the language of mathematics is the gold standard, modeled after physics. In physics, the laws of natures are couched in the mathematical descriptions in the form of differential equations, known as the mathematical models. Much work has been done in the West to extend such methods of physics to the study of medicine. One such example is the science of system biology where the tools of systems and control theory, which grew out of cybernetics, are* applied* to study bio-organisms. There are, however, inherent limitations.

The dynamics of bio-organisms are, by nature, complex, uncertain, always in flux, and individually unique. A mathematical model is, at best, a snapshot of a particular organism at a particular time, under a particular set of circumstances. Even so, it is still a rough approximation. It is therefore evident, even beyond doubt, that the modeling and model-based approach to bio-organisms are fundamentally limited. The solution in quantifying the TCM appears to lie elsewhere, as discussed in this section.

### 3.1. The Cybernetic Model of Wuxing

The TCM is consistent with the unique cosmic view of Yin-Yang and Wuxing. Out of the primordial soup of uniformity, of the one (Yuanqi), of the indistinguishable, came the Yin and Yang for which Wuxing is the further delineation and manifestation, as in nature and as in human body. The TCM, rooted in Wuxing, holds human body as an organic whole that consists of five Zang viscera and six Fu viscera; the* qi*-blood and the bodily fluid; the meridian; the body constituents, sense organs and ortifices; and the limbs and other parts. Each viscus has its own outside signs and is spatially related to certain body constituents and orifices. The five Zang viscera, including the liver, heart, spleen, lung, and kidney, are different in their physiological functions and pathological changes, but they are interrelated, that is, interpromoting, interrestricting,and interaffecting. It is these relationships among the five Zang viscera that are crucial to the working of the human body as a whole.

Physiologically, the TCM further stipulates that the functions of various visceral tissues and organs can be classified into the systems of five Zang organs, respectively, by the analogy to the five elements in Wuxing. Specifically, anchored in the five Zang organs are five distinct physiological systems that are interconnected through the meridian and extended to the six Fu organs of the gallbladder, stomach, small intestine, large intestine, urinary bladder, and Tri-Jiao. These five systems based on the five Zang organs in human body mirror the inner working of nature as seen in the cybernetic model of Wuxing, reflecting the mutuality between human and nature as seen in the Chinese philosophy.

In short, the manifestation of the Wuxing model of nature in human body gives us the five interconnected systems symbolized by the five Zang organs. The five elements in Wuxing may be seen as state variables in some sense; the medical treatments and medicines are control actions; the internal and external anomalies in the human body can be seen as disturbances, and the diagnosis based on the TCM produces the output measurements. And, just like in nature, each of the five systems of human body exerts influences on other systems and, in turn, is influenced by them; they are both controlling and are controlled at the same time, forming an intricate chain of causality that challenges the mind of the best of us. The impact of one system on the other can be seen as promoting (enabling) or restricting (inhibiting), directly or indirectly. In engineering terms, the “feedback” among the five systems can be seen as positive (reinforcing) or negative (limiting) and it is the collective whole of such relationships that determines the Yin-Yang balance in the human body.

When Tsien calls cybernetics a science of interrelations [4], perhaps he is influenced, knowingly or not, by Wuxing. When he described the feedback control of the turboalternator as a way to achieve the energy balance, perhaps he saw the connection of what N. Wiener described as control and communication in the animal and the machine. In any event, perhaps the time has finally come to adopt the thinking of engineering cybernetics of Tsien as a quantitative means to the holistic science of cybernetics and the TCM.

### 3.2. The Cause of Sickness: The Imbalance of the Body

The science of the causes and effects of diseases, that is, pathology, is the basis of medicine, Eastern or Western. The cybernetic model of Wuxing offers a distinctly different perspective, that is, complementary to the existing model based on modern science. The sickness, in view of Wuxing, is necessarily accompanied by certain imbalance in the body. In other words, the cause of disharmony leads to the ailment which can be seen as an undesirable* state* of the body. In the context of the theory of dynamic systems, the changes in the state are governed by the principle of causality [17], which tells us that the state is determined by the initial value and the external forces known as inputs. It is those harmful external forces, that is, disturbance, which is also known as* xie* or pathogenic* qi* in the TCM, that cause the imbalance in a body and the corresponding ailment, if any. The Western medicine focuses on the material basis of such disturbances, such as bacterial infection or dysfunctions of certain organs; the cybernetic model of Wuxing emphasizes the mutual relationships among the five systems in the body.

The ideal state of a body in the Western medicine is one that is disease-free; in the TCM, however, it is the perfect harmony among the five life systems symbolized by the five Zang organs of liver, heart, spleen, lung, and kidney. In other words, the ideal body in the TCM is one that the promotions and restrictions, or the positive and negative feedbacks, among the five life systems are perfectly balanced. The disturbances are powerless against such bodies. Such bodies may be seen in the Western medicine as having strong immunity against diseases, but it is the cybernetic model of Wuxing that lays the foundation for further scientific study and medical practice. Perhaps this is a place where the TCM and the Western medicine find the common goal, amid different philosophical orientations.

The imbalance in the body, according to Wuxing, is caused by the anomalies in the relationships, both promotional and restrictive. Those happened in the former are called mother-child involvement (MCI), where those who promote and those who are promoted are mutually affected; those happened in the latter are known as subjugation and violation. Subjugation refers to an abnormal condition in which one of the five elements excessively restricts another element; violation refers to an abnormal condition in which one of the five elements reversely restrains and bullies the element that normally restricts it. Such anomalies caused by various small disturbances are not common, nor are they serious because, in the view of Wuxing, the mutual relationships among all five systems are self-balancing and self-repairing. In such cases, no external intervention, that is, adjustment or treatment, is needed.

In the presence of large and sustained disturbances, however, some or all of the five systems will be weakened and self-restoration becomes increasingly difficult. Li [18] from the Ming dynasty points out that “when the disease causing agent (*xie*) first entered the body, the positive* qi* is still strong and its opposite is weak, and the proper strategy is to attack the* xie*; if the* xie* persists and its effect deepens, the positive* qi* declines and the proper strategy is to both attack the* xie* and strengthen the body; finally, if and when the disease reaches the final stage where* xie* is ubiquitous and the positive* qi* is exhausted, the only remedy left is to invigorate the body. Therefore, the problem of ailment is reduced to that of balance, which is then linked to the problem of disturbance. It is here that the engineering principle of disturbance rejection can be used to clarify the principle and practice of the TCM.

### 3.3. Active Disturbance Rejection

The connection illustrated above between the TCM and the engineering principle of disturbance rejection makes relevant many engineering methods developed over the last few decades. In this section, a particular method of ADRC is used as an illustration.

Human body can be seen, in engineering terms, as a dynamic system, that is, bombarded constantly by disturbances of various kinds. Treatment, as external intervention, is deemed necessary when the body is out of balance and enters into an undesirable state, manifested in illness. The reduction or elimination of deviations from the ideal state is the common goal in medicine and in engineering.

ADRC, as introduced earlier, is a unique engineering solution to disturbance problem in that it seeks to actively reject the disturbance, as oppose to passively respond to its consequence. Its principle is strikingly similar to the basic tenant of the TCM: the supreme doctor treats the disease, that is, yet to occur. That is, a doctor with superior skills and understanding of the TCM will be able to detect and treat the subtle imbalances before they manifest themselves as ailments. The practice of the TCM medical acts as an outer loop to the control system, that is, already there in the body, helping the body to go back to the equilibrium through external assistance. In ADRC, such imbalance is detected early using the tools of the extended state observer, which extracts the disturbance information from the input-output data of the physical process. In the TCM, such information is obtained through four kinds of diagnosis means: watching, smelling, interrogating, and feeling of the pulse. In both ADRC and the TCM, the emphasis is on seeing early the cause of imbalance so as to minimize its effect. In other words, the disturbance rejection paradigm and its product in ADRC are the engineering equivalent of disease prevention and strengthening of the autoimmune system in human body.

In the cybernetic model of Wuxing and ADRC, we found a language to articulate the principles and methods of the TCM. The full exposition of it is of course beyond the scope of a single paper, but the main idea can be seen with an illustrative example, as shown below. It concerns with the treatment of liver cancer based on the interrelationships between the liver system and other systems of Wuxing. In particular, the promotion and restriction relationships around liver system provide the guideline for medical intervention, the effectiveness of which is demonstrated.

## 4. Case Study

In this section we demonstrate, by example, how the cybernetic model of Wuxing can be used to establish the therapeutic principles and methods in the treatment of certain liver cancer. A disease in any Zang organ, according to Wuxing, will propagate up and downstream in both the promotional and restrictive relationships to other organs, causing imbalance in this interconnected system of Wuxing. The treatment, therefore, must be holistic and not limited to the dysfunctional organ.

In particular, among the promotional relationships are the therapeutic principles of tonifying the mother and letting go of the child: the former rectifies the deficiency in the mother-child relationship in Wuxing and the latter reduces the excess. Typical treatments in the promotional relationships include replenishing water to nourish wood, mutual promotion of metal and water, reinforcing earth to strengthen metal, and fueling fire to strengthen earth. The corresponding material means will be explained shortly in the example.

Furthermore, among the restrictive relationships are the therapeutic principles of checking the strong and strengthening the weak: the former is a therapeutic principle to restrict the hyperactive viscus so as to benefit the recovery of the subjugated or violated viscus; the latter is to enrich the subjugated or violated viscus so as to balance the strengths of both sides. This applies to the case where the power for restriction becomes weak due to subjugation or violation. Typical treatments in the restrictive relationships include inhibiting wood to assist earth, banking up earth to treat water, assisting metal to subdue wood, reducing the south, and tonifying the north.

The application of these therapeutic principles and methods based on the cybernetic model of Wusing is shown below in an example based on the results from a sequence of liver cancer treatment studies.

### 4.1. Liver Cancer: Pathogenic Factors and Pathogenesis

Pathogenic factors of liver cancer are complex, in view of the TCM, and they are associated with stagnation of* qi* from sustained low mood and the lack of adequate flow; the dysfunction of the Zang and Fu organs resulting in inadequate cleansing of the turbidity; and the inadequate flow of* qi* and blood, leading to accumulation syndrome, that is, fiery and poisonous. Therefore, the TCM concludes the liver cancer is a disease of amassing in the abdomen, of tympanites, and of jaundice, and so forth.

As the liver encounters the pathogenic* qi*, it rises up and attacks. Both the healthy and the pathogenic* qi* are exuberant at this stage and their struggle is violent and sustained. As the pathogenesis develops, the pathogenic* qi* takes hold and the healthy* qi* degrades, leading to the dysfunction of the liver and the symptoms of difficulty of dispersing,* qi* stagnation, phlegm obstruction, improper* qi* accumulation, lack of blood movement, and the gathering syndrome. As the pathogenic* qi* becomes increasingly stronger, the disease heightens, so does the loss of the healthy* qi*, until it is mostly gone, along with any resistance to the disease, which finally reaches the terminal stage.

In view of the cybernetic model of Wuxing, a human body is a dynamic system with mutually dependent five Zang systems. The above process of pathogenesis can be understood in the framework of disturbance rejection: the liver system is affected by disturbances and loses balance; the disturbance is compensated by the use of healthy* qi* which has limited supply. When such imbalance is not restored quickly, due to the severity of the disturbance and the weakened state of the body, the liver system runs out of energy and the disturbance rejection ability. At this point, only the external intervention, based on the understanding of the cybernetic model of Wuxing, can help the system recover its balance. The question is what Wuxing can tell us.

Wuxing tells us that even though the cancer is located at the liver, the treatment must include those organs that are mutually dependent on it, which in this case are the spleen and the kidney systems. The spleen has the restrictive and the kidney has the promotional relationships with the liver, respectively. The contents of these complicated relationships are explained as follows.

The cause of the liver cancer and its development are quite complicated. It could be the liver itself or the complications from other mutually dependent organs. The pathogenesis centers on the dysfunction of the liver, and it initially leads to the* qi* stagnation, ecchymosis, and fluid retention, and the interplay of the three takes hold in the liver and manifests itself in a variety of symptoms. Gradually, this condition propagates to the spleen and kidney and causes the spleen deficiency and the kidney impairment, while the liver itself loses the function of blood storage. At the later stage, the pathogenesis propagates to the heart system, leading to sudden unconsciousness, convulsive syncope, and hemorrhage [19, 20]. Based on such understanding of pathogenesis rooted in Wuxing, the treatment is devised accordingly, similar to the principle of active disturbance rejection in engineering [16].

### 4.2. The Wuxing-Based Therapeutic Approach

The cybernetic model of Wuxing provides the guideline, the principles, and the methods of a unique treatment of liver cancer. Specifically, the following herb medicines are used: the Jiang Huang (Rhizoma* Curcumae longae*) that enters the liver meridians and activates the blood and moves the* qi* and the Huang Qin (*Radix Scutellariae*) that enters the meridians of the lung, the gallbladder, and the large intestine for its functions of removing heat and dry dampness and purging the fire and toxicity. The main ingredient of Huang Qin is baicalin.

Because, according to Wuxing, the lung has the property of being mental and it restricts the liver. Lessening such restriction on the liver can be accomplished by using Huang Qin to first relieve lung and to dampen it a bit, as a precaution. That is, the Wuxing foretells the weakening of the liver by the restriction from the lung and this can be countered beforehand by first relieving the lung.

Heart, on the other hand, has the property of fire and is promoted by the liver. The dysfunction of the liver will likely to cause excessive fire and this can be, again, countered beforehand using the herbal medicine of Mu Dan Pi (Cortex Moutan), of which the main ingredient is Paeonol. It enters into the meridians of the heart, the liver, and the kidney, removing the heat, cooling and activating the blood, and resolving the stasis. Chuan Xiong (Rhizoma Chuanxiong) is another useful herbal medicine with the main ingredient of tetramethylpyrazine, which enters the meridians of the liver and the pericardium with the functions of activating the blood, moving the* qi*, suppressing the wind, and alleviating the pain. The preventive use of these two herbs is guided by the Wuxing's mother- (liver) child (heart) relationship discussed above. In anticipation of the liver's problematic behavior, the heart is first relieved before the excessive fiery effect from the liver arrives. In other words, to remove heat/fire generated by a malfunction liver, both the liver and the downstream heart are treated by the blood activating and* qi* moving medicine for better transport of the heat and fire.

The same principle of Wuxing and disturbance rejection also applies to spleen which is restricted by the liver. At the initial stage of the disease when the liver's activities are excessive, the spleen will likely be exceedingly suppressed. To counter such disturbance, reinforcement should be applied, again, beforehand, such as the use of Shan Yao (*Rhizoma dioscoreae*), which enters into the spleen, lung, and kidney meridians; fortifies the spleen and stomach; promotes the production of bodily fluid to nourish the lung; and strengthens the kidney to arrest spermatorrhea.

Note that the promotion of the liver and strengthening of the spleen can be carried out simultaneously by using the combination of Huang Qin and Shanyao.

Finally we arrive at the kidney, the most important organ in Wuxing, which has the property of water and promotes the liver. The corresponding treatment method is called replenishing the water to nourish the wood, using the herbal medicine of Nv Zhen Zi (Fructus Ligustri Lucidi), which enters the meridians liver and kidney and nourishes them. The second herbal medicine is Gou Qi Zi (*Fructus lycii*) which also enters the meridians of the liver and kidney and nourishes them. Both medicines also have the benefits of promoting eye sight, which is closely related to the health of the liver. Because of the mother-child relationship between the kidney and the liver, the Yang of the liver is astringed by enhancing the kidney.

### 4.3. Results from the Experimental Studies

A series of experimental studies has been carried out in the last six years to systematically test the therapeutic treatment based on the cybernetic model of Wuxing and the engineering concept of disturbance rejection. These experiments are designed to test if the results agree with the theory, using the herbal medicine discussed above, including curcumin, ursolic acid, baicalin, paeonol, tetramethylpyrazine, as well as the combination of curcumin and* Rhizoma dioscoreae*, and the combination of ursolic acid and* Fructus lycii*. The treatments were applied to the precancerous lesion of mice liver, induced by using the diethylnitrosamine (DEN) [21–24], a standard method.

The effects of the curcumin and* Rhizoma dioscoreae* combination were first examined by the liver histological analysis and activities of serum marker enzymes. The experimental results show that DEN initiation led to a remarkable increase of serum marker enzymes, and abnormality such as bile canaliculi hyperplasia and presence of tumor cells were observed in liver histopathological examination in the model mice, while the control ones revealed the normal architecture.

Oral treatment of curcumin and the treatment of the curcumin and* Rhizoma dioscoreae *combination resulted in a marked reduction in serum marker enzymes and improvement in liver histopathology compared with the model ones. The conclusion was that curcumin can protect the hepatic precancerous lesions in the mice attacked by diethylnitrosamine, and combination use of curcumin and* Rhizoma dioscoreae* can be more effective [16, 25]. This shows the effectiveness of the method of inhibiting the wood and replenishing the earth discussed above. Moreover, it demonstrates that treating the liver and, at the same time, nourishing the spleen greatly enhanced the treatment, just as the cybernetic model of Wuxing and the active disturbance rejection principle suggested.

Similarly, the method of nourishing both liver and kidney is also put to test by using the ursolic acid from* Fructus ligustri lucidi* and by combining ursolic acid with* Fructus lycii*. Results show that both treatments lead to improvements but the latter is better [26]. This confirms the method of replenishing the water to nourish the wood, based on the Wuxing and the disturbance rejection principles.

Later experiments show that tetramethylpyrazine, paeonol, and baicalin were partially effective to protect liver from the carcinogenesis initiated by DEN; tetramethylpyrazine and paeonol both activate the blood and play the role of relieving the child in the cybernetic model of Wuxing. The readers are referred to [27–29] for more detailed results and other related studies.

In summary, the pathogenesis of the liver cancer is complex because of the mutually dependent relations among all five Zang organs. The treatment of the liver must be accompanied by the treatment of other organs according to the cybernetic model of Wuxing and the principle of disturbance rejection.

## 5. Conclusions

In this paper we attempt to establish a framework for the study of the TCM where a new language of engineering cybernetics is introduced to delineate, with clarity, the principles and practice of the TCM. In engineering cybernetic we find a holistic view, a science of interrelations, with strong resemblance to what the Wuxing attempts to do: describing the inner workings of nature and human body alike as mutually connected components, that is, closed-loop dynamic systems. EC and the TCM also share a commitment and goal to attain and maintain a quality, that is, described as balance, equilibrium, or homeostasis; correspondingly there is a great similarity in how such quality is maintained, by what is called “disturbance rejection” in EC. The engineering invention of active disturbance rejection shares the common goal with the TCM in anticipating and rejecting the disturbances before the balance is disrupted. Combining the vocabulary of EC and the TCM we found the cybernetic model of Wuxing as the common language and a promising new path towards the quantitative study of the TCM. As an initial study and illustration, the pathogenesis and treatment of the liver cancer based on the TCM are explained using the language of the cybernetic model of Wuxing and the principles of disturbance rejection. The results from a six-year study seem to support this line of investigation and to provide a clear exposition of both the principles of the TCM and how they are practical with herbal medicine.

Human body is a complex, large, and open system that defies our complete grasp. It has always been a great challenge to organically combine the best offerings from the Western medicine and the Eastern medicine, largely due to the lack of common language. In EC we find the language of a holistic science for the West, that is, inherently congenial to the Eastern thinking in Yin-Yang and Wuxing. It is our hope that what started here could help future researchers to build the bridge from the general system qualities and behaviors to the properties of the system components from the qualitative to the quantitative and from the abstract concepts to the concrete practice.
